# *In silico* and *in vivo* verification of the mechanism of formononetin in treating hepatocellular carcinoma

**DOI:** 10.1080/07853890.2024.2404550

**Published:** 2024-09-20

**Authors:** Guiping Ma, Xu Pang, Yun Ran, Wenlin Chen, Yichi Zhou, Xiaobin Li, Bowen Liu, Feng Li, Shiping Hu

**Affiliations:** a Beijing University of Chinese Medicine Affiliated Shenzhen Hospital, Shenzhen, China; b Beijing University of Chinese Medicine, Beijing, China

**Keywords:** Formononetin, hepatocellular carcinoma, network pharmacology, molecular docking, *in vivo* experiment

## Abstract

**Background:**

Hepatocellular carcinoma (HCC) remains a significant global medical challenge. Formononetin, an isoflavone derived from *Astragalus membranaceus*, has been shown to have various regulatory effects on HCC. However, the exact molecular mechanism by which formononetin acts against HCC is still unclear.

**Purpose:**

To elucidate the molecular mechanism of formononetin in treating HCC.

**Methods:**

The potential targets of formononetin were retrieved from Swisstargets and SEA databases, while targets associated with HCC were sourced from GeneCards, NCBI and DisGeNET databases. The overlapping targets were visualized using protein–protein interaction (PPI) network analysis via String database, and subsequently subjected to Gene Ontology (GO) and Kyoto Encyclopedia of Genes and Genomes (KEGG) enrichment analysis. Molecular docking was employed to confirm the interaction between formononetin and key targets. Ultimately, the effectiveness of formononetin on HCC and the signalling pathway with the highest enrichment were confirmed in the HCC tumour-bearing mice. Histopathological changes in tumour tissues were observed using haematoxylin and eosin (HE) staining, while apoptosis of tumour cells in mice was assessed through TdT-mediated dUTP nick end labelling (TUNEL) and immunofluorescence staining. The most enriched signalling pathway was verified using Western blotting and immunohistochemical (IHC) staining.

**Results:**

One hundred and ninety-three potential targets related to formononetin, 6980 targets associated with HCC and 156 overlapping targets were obtained from the online public databases. Molecular docking studies demonstrated formononetin’s robust interaction with core targets. KEGG enrichment analysis identified 111 signalling pathways, including PI3K/AKT and apoptosis signalling pathways. *In vivo* experiments demonstrated that formononetin significantly promoted apoptosis of tumour cell in mice, as confirmed by HE, TUNEL and immunofluorescence staining (*p* < .05). Formononetin was found to decrease the phosphorylation levels of PI3K and AKT, reduce the expression of Bcl-2, and increase the expression of cleaved-Caspase-3 and Bax (*p* < .05).

**Conclusions:**

Formononetin demonstrates dose-dependent regulatory effects on multiple targets, biological processes and signalling pathways in HCC. The compound can mitigate HCC by enhancing PI3K/AKT-mediated apoptosis of tumour cells.

## 1. Introduction

Primary liver cancer is the third leading cause of cancer-related death worldwide (*n* = 830,180; 8.3%) and represents a major threat to the health of people around the world [1,2]. The pathological types of primary liver cancer are divided into hepatocellular carcinoma (HCC), bile duct cell carcinoma and mixed liver cancer. Among them, HCC accounts for around 90% of primary liver cancer [3,4], which is the most common type. HCC is a malignant invasive epithelial cell tumour that originates from liver cells, and usually occurs in the case of chronic viral hepatitis and cirrhosis. Clinical management of HCC mainly includes surgical and non-surgical treatment. Unfortunately, only 5–15% of early-stage HCC patients are suitable for surgical resection, which carries a 5-year survival rate of 70% [5]. Most patients with HCC are usually diagnosed at a late stage, resulting in a poor prognosis. Currently, the treatment of HCC is mainly long-term use of chemotherapy drugs, which is prone to adverse effects such as drug resistance and toxicity. Therefore, it is of great significance to explore new anti-cancer targeted treatment drugs for HCC-related signalling pathways.

Accumulating studies have shown that a variety of natural compounds in traditional Chinese medicine play a role in inhibiting mechanisms related to cancer development. These compounds can provide new cancer treatment solutions by activating anti-tumour, anti-proliferation and antioxidant systems [6–8]. To improve the efficacy of HCC, it is particularly important to extract active anticancer natural products from traditional Chinese medicine. *Astragalus membranaceus* is one of the traditional Chinese medicines and is listed in the Chinese Pharmacopoeia. In recent years, with the continuous deepening of research on *Astragalus membranaceus*, it has been found to have many pharmacological effects, among which the study of the anti-tumour effect of *Astragalus mem­branaceus* has attracted widespread attention [9]. Formononetin is an isoflavone extracted from *Astragalus membranaceus*, has been reported to exhibit anti-tumour activity by promoting apoptosis of tumour cells [10,11] and inhibiting cell proliferation and invasiveness [12]. Previous study reported that the inhibitory effect of formononetin on HCC cell proliferation was related to hepatocyte metabolism and cell cycle regulation-related pathways [13], but the mechanism of formononetin against HCC is still not completely clear.

**Figure 1.**
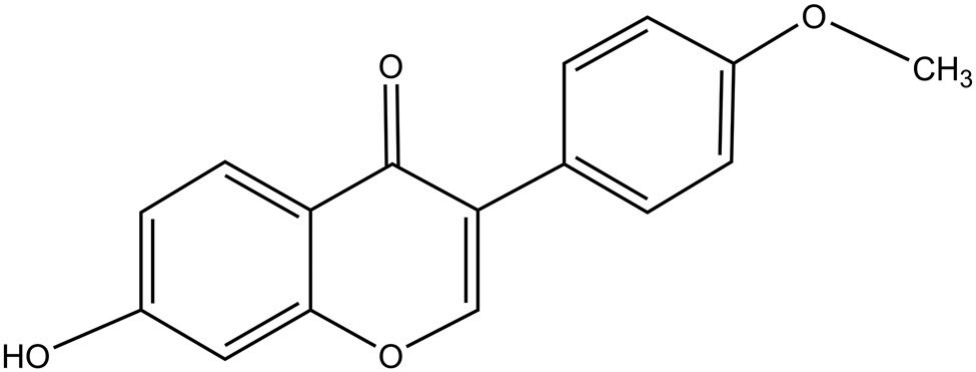
The chemical structure of formononetin.

Therefore, in this study, a comprehensive strategy of *in silico* and *in vivo* experiments was used to explore the underlying molecular mechanism of formononetin (Figure 1) in HCC mouse models.

## 2. Materials and methods

### 2.1. Acquisition of overlapping targets between formononetin and HCC

Initially, the SMILES ID of the formononetin was acquired from PubChem database (https://pubchem.ncbi.nlm.nih.gov/), followed by importing into the Swisstargets (http://www.swisstargetprediction.ch/) and Similarity ensemble approach (SEA) databases (http://sea.bkslab.org/) to identify the associated compound targets. Subsequently, the compound targets of the formononetin were explored utilizing the Pharmmapper (http://www.lilab-ecust.cn/pharmmapper/) and traditional Chinese medicine systems pharmacology (TCMSP) databases (https://tcmspw.com/tcmsp.php). The identified targets were validated and de-duplicated using the Universal protein (UniProt) database (https://www.uniprot.org/). Then, the term ‘hepatocellular carcinoma’ was employed as a search keyword to retrieve human genes from the GeneCards (https://www.genecards.org/), NCBI and DisGeNET databases (https://www.disgenet.org/). Targets sourced from the GeneCards database were refined based on their median score to prioritize more pertinent targets. A Venn diagram analysis was then conducted to illustrate the shared targets between HCC and formononetin.

### 2.2. Construction of the protein–protein interaction network

The identification of core regulatory genes can be facilitated through the analysis of protein–protein interactions (PPIs). In this study, PPI analysis was conducted using the String database (https://string-db.org/cgi/input.pl) with a focus on the species ‘Homo sapiens’ and a filtering of interaction scores >0.4. Nodes with higher degrees were identified as the core targets.

### 2.3. Construction of component–disease–target network

To enhance comprehension of the intricate interplay between formononetin, HCC and their respective targets, we developed a component–disease–target network utilizing these entities and subsequently imported it into Cytoscape 3.8.0 for visualization.

### 2.4. GO and KEGG pathway enrichment analysis

To elucidate the functions of core targets and investigate their associated biological processes (BPs) and signalling pathways, an enrichment analysis was conducted on the core targets. The analysis included an examination of the BP, molecular function (MF) and cell component (CC) enrichment using Gene Ontology (GO). Additionally, a Kyoto Encyclopedia of Genes and Genomes (KEGG) pathway enrichment analysis was conducted on the overlapping targets between formononetin and HCC (www.kegg.jp/kegg/kegg1.html). Targets with corrected *p* values <.05 were identified. The software packages Cluster Profiler, enrich plot and ggplot2 were utilized in R 4.0.3 software to generate bar and bubble plots.

### 2.5. Construction of component–disease–pathway–target network

The network file containing information on components, diseases, pathways and targets was imported into Cytoscape 3.8.0 to generate a pathway network diagram. This visualization allowed for a more intuitive demonstration of the multi-component and multi-target functions of active components in disease treatment.

### 2.6. Molecular docking

The formononetin molecules were subjected to molecular docking with core targets exhibiting higher degrees of interaction, as identified through PPI network analysis. The 3D structures of the molecules in SDF format were retrieved from PubChem data and imported into ChemBio3D Ultra 14.0 for energy minimization, with the minimum RMS gradient set to 0.001. The resulting small molecules were saved in mol2 format. Subsequently, the molecular structures of the core targets were queried in the RCSB Protein Data Bank (PDB) database (https://www.rcsb.org/). The ligands and nonprotein molecules present in the core targets, such as water molecules and primitive ligands, were removed using Pymol 2.3.0 and saved in PDB format. POCASA 1.1 was employed for the prediction of protein binding sites, while AutoDock Vina 1.1.2 was utilized for docking. Additionally, the interaction pattern of the docking results was analysed using Discovery Studio 2019.

### 2.7. Experiment verification

#### 2.7.1. Cell culture

The H22 cell line was procured from Wuhan Bena Technology Co., Ltd. (Wuhan, China) and maintained in a 37 °C, 5% CO_2_ incubator with RPMI-1640 medium supplemented with 10% foetal bovine serum (FBS; Gibco, Pleasantville, NY), 100 units/mL penicillin and 100 μg/mL streptomycin (Gibco, Pleasantville, NY).

#### 2.7.2. Animals, model construction and grouping

The animal experiments conducted in this research were ethically approved by the Animal Experiment Ethical Committee of Beijing University of Chinese Medicine affiliated Shenzhen Hospital (No. KY-2023-047) and adhered to the Reporting *In Vivo* Experiments (ARRIVE) guidelines. Formononetin (HY-N0183, purity of 99.92%) was purchased from MedChemExpress Biotech (MCE, Monmouth Junction, NJ). Fifty male Kunming mice, aged 6 weeks and weighing 20 ± 2 g, were procured from Guangdong Medical Laboratory Animal Center (Foshan, China). Mice were housed in a controlled environment free of specific pathogens, maintained at 25 ± 1 °C, and provided free access to food and water. After a seven-day adaptive feeding period, mice were inoculated with H22 cells (1 × 10^7^) in the left axilla to establish HCC models. The successful establishment of the HCC model was confirmed by the palpable formation of tumours approximately 5–7 days after inoculation. In accordance with the random number table, the mice were randomly subdivided into model group (MOD, *n* = 10), sorafenib group (SRFB, *n* = 10), low-dose formononetin group (L-FMNT, *n* = 10), medium-dose formononetin group (M-FMNT, *n* = 10) and high-dose formononetin group (H-FMNT, *n* = 10). The FMNT groups and positive control group were orally administered formononetin (50, 100 or 200 mg/kg/d) or SRFB (30 mg/kg/d). Meanwhile, the mice in model group were orally administered the same volume of 0.9% saline. The mice were euthanized following 14 days of uninterrupted intervention, and the tumour tissues were subsequently harvested for additional analysis.

#### 2.7.3. Histological analysis

The tumour tissues were fixed in 4% paraformaldehyde for 48 h, embedded in paraffin and sliced into 4 μm sections. Histopathological morphology of the tumour tissues of mice was evaluated by haematoxylin and eosin (HE) staining. Immunohistochemical (IHC) staining was performed on tumour tissue sections to detect the protein expression of cleaved-Caspase3 (25128-1-AP, 1:100, Proteintech, Wuhan, China). The images of sections were photographed using a light microscope.

#### 2.7.4. TUNEL staining

TdT-mediated dUTP nick end labelling (TUNEL) staining was applied to assess the apoptosis of tumour cells within each experimental group. Following dehydration in 30% sucrose for 24 h, the tumour tissues were washed and subsequently embedded in paraffin. Subsequent staining procedures were conducted in accordance with the manufacturer’s instructions of the TUNEL kit, and resultant images were visualized and captured utilizing a fluorescence microscope.

#### 2.7.5. Immunofluorescence

Immunofluorescence analysis was performed on paraffin-embedded skin tissue sections. For antigen repair, the sections were placed in 0.1 mol/L citric acid repair solution (pH = 6), heated in a microwave oven for 6 min to slightly boil, maintained in a medium heat for 10 min and naturally cooled for 20–30 min. After washing with PBS, the membranes were permeabilized with 0.2% triton X-100 for 20 min. Washing with PBS, adding blocking serum and blocking the sections in a wet box for 1 h at room temperature. Washing with PBS, adding antibody B-cell lymphoma-2 (Bcl-2) (1:200, 26593-1-AP, Proteintech, Wuhan, China) and incubating in a wet box at 4 °C overnight. The sections were rewarmed for 30 min and eluted three times with PBS. Fluorescent secondary antibodies were incubated for 2 h at room temperature, carefully protected from light. The nuclei were stained with DAPI, and fluorescence images were obtained by fluorescence microscope.

#### 2.7.6. Western blotting analysis

The protein concentration of the supernatant was measured using a BCA Protein Assay Kit. The proteins were boiled at 95 °C for 10 min and stored at −20 °C after being converted into equal quality and volume. SDS-PAGE gels (10%) were used to electrophorese the proteins, which were then transferred onto PVDF membranes in an ice bath at 350 mA for 60 min. The membranes were blocked with 5% skim milk at room temperature for 1 h and incubated with primary antibodies and secondary antibodies. The following antibodies GAPDH (60004-1-Ig, 1:1000), PI3K (20584-1-AP, 1:1000), AKT (60203-2-Ig, 1:1000), phospho-AKT (66444-1-Ig, 1:1000), Bcl-2 (26593-1-AP, 1:1000), Bax (50599-2-Ig, 1:1000), cleaved-Caspase3 (25128-1-AP, 1:1000) and Caspase3 (66470-2-Ig, 1:1000) were purchased from Proteintech (Wuhan, China). The antibody phospho-PI3K (AP0854, 1:1000) was obtained from Abclonal (Wuhan, China). The anti-mouse IgG (7076, 1:3000) and anti-rabbit IgG (7074, 1:3000) were obtained from Cell Signaling Technology, Inc. (Beverly, MA). Finally, the protein bands were visualized using Image Lab software and analysed using Image J (1.8.0) software (Bethesda, MD).

#### 2.7.7. Statistical analysis

All data are presented as the mean ± standard deviation. SPSS 26.0 software (SPSS Inc., Chicago, IL) was used to analyse the data. All histogram data are expressed as the mean ± standard error of the mean (SEM). A one-way analysis of variance (ANOVA) and Tukey’s multiple comparison test were used for multiple group comparisons. Comparative differences were considered statistically significant at a *p* value of <.05.

## 3. Results

### 3.1. Candidate targets of formononetin against HCC

**Figure 2.**
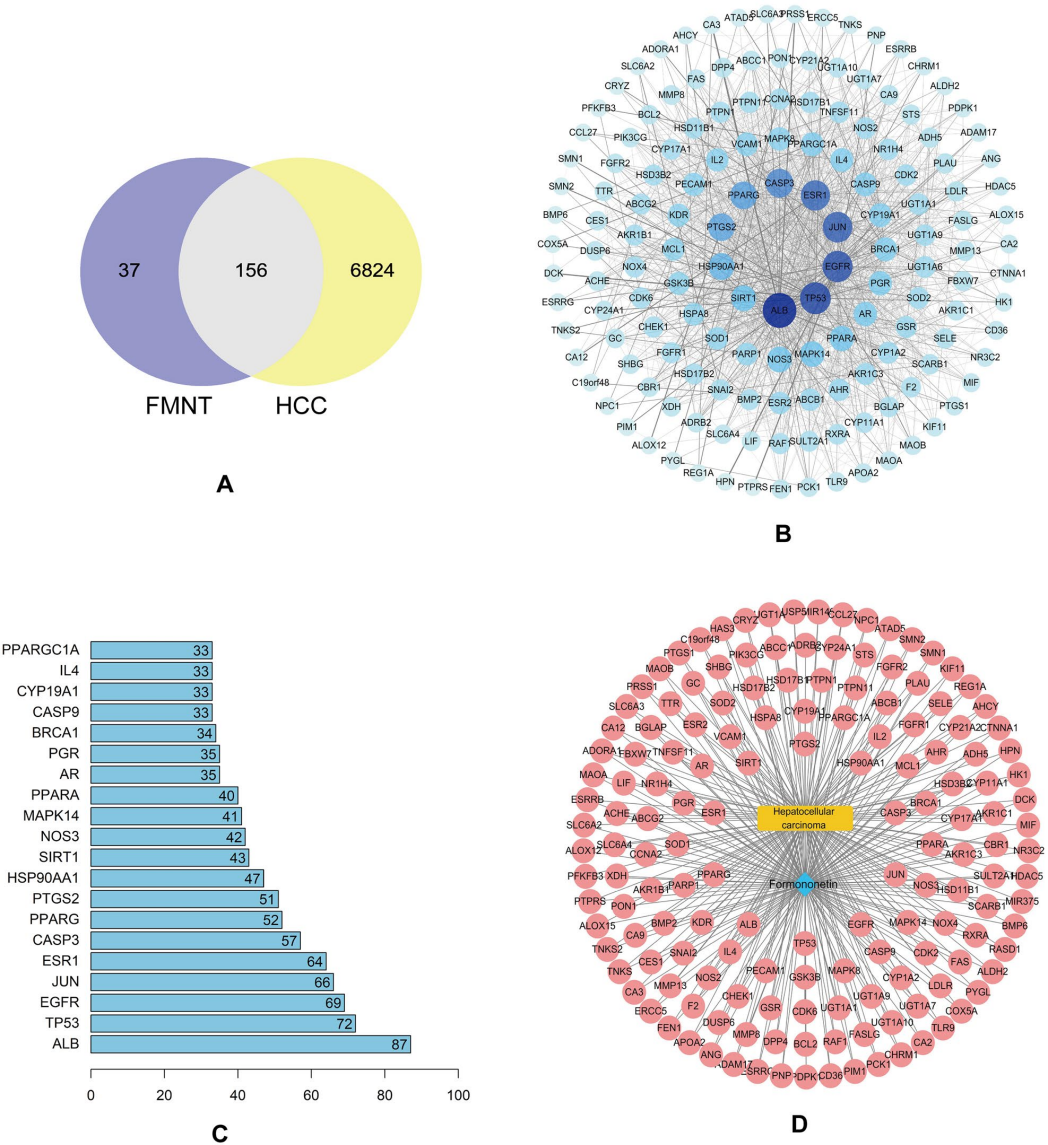
The common targets and PPI network of formononetin against HCC. (A) Venn diagram of formononetin targets and HCC-related genes. (B) PPI network of formononetin against HCC. (C) The top 20 targets in PPI network. (D) Formononetin–potential target genes–HCC network. The blue node represents formononetin; the red node represents potential targets; and the yellow represents HCC.

As shown in Table 1 and Figure 2(A), a total of 6980 HCC-related targets and 193 formononetin-related potential targets were obtained from the online databases. Moreover, 156 overlapping targets of formononetin treating HCC were acquired using Venn diagram analysis. These candidates may be the potential therapeutic targets of formononetin treating HCC. Formononetin exhibited a significant intervention effect on HCC due to most of its related targets being potential targets for the treatment of HCC.

**Table 1.** Potential targets of formononetin in the treatment of HCC.

No.	Potential targets	No.	Potential targets	No.	Potential targets	No.	Potential targets
1	ESR1	40	HSP90AA1	79	JUN	118	IL4
2	PIM1	41	ALDH2	80	PIK3CG	119	SIRT1
3	ESR2	42	HK1	81	MCL1	120	PTGS1
4	CES1	43	HPN	82	AHR	121	ACHE
5	CDK2	44	CTNNA1	83	FBXW7	122	MAOB
6	AR	45	AHCY	84	MIR149	123	PRSS1
7	CA2	46	CDK6	85	SOD1	124	RXRA
8	ALB	47	AKR1C3	86	USP5	125	ADRB2
9	STS	48	SULT2A1	87	SNAI2	126	GSK3B
10	APOA2	49	ADH5	88	PPARG	127	SLC6A3
11	PGR	50	MAPK8	89	PPARA	128	NOS2
12	MMP13	51	AKR1C1	90	CYP17A1	129	SLC6A4
13	HSPA8	52	SOD2	91	CYP11A1	130	CHRM1
14	F2	53	PLAU	92	CYP24A1	131	IL2
15	DUSP6	54	PTPN11	93	PPARGC1A	132	CA12
16	ANG	55	REG1A	94	VCAM1	133	ADORA1
17	MMP8	56	KDR	95	CYP21A2	134	MAOA
18	ADAM17	57	NR1H4	96	HSD3B2	135	ESRRB
19	DPP4	58	KIF11	97	LIF	136	ABCG2
20	CHEK1	59	AKR1B1	98	SELE	137	HSD17B2
21	MAPK14	60	TP53	99	PECAM1	138	CBR1
22	PYGL	61	SMN1	100	UGT1A	139	SLC6A2
23	PDPK1	62	SMN2	101	LDLR	140	ALOX12
24	GSR	63	UGT1A1	102	PARP1	141	XDH
25	ESRRG	64	UGT1A9	103	BCL2	142	PFKFB3
26	PNP	65	UGT1A10	104	FAS	143	PTPRS
27	FGFR1	66	RASD1	105	ABCC1	144	ABCB1
28	EGFR	67	BRCA1	106	CASP3	145	ALOX15
29	SHBG	68	ATAD5	107	TNFSF11	146	TLR9
30	HSD17B1	69	UGT1A7	108	CYP19A1	147	NOX4
31	HSD11B1	70	NPC1	109	FASLG	148	PON1
32	NR3C2	71	PTGS2	110	SCARB1	149	TNKS2
33	GC	72	FGFR2	111	CASP9	150	TNKS
34	TTR	73	MIR375	112	CYP1A2	151	CA3
35	PCK1	74	HDAC5	113	BMP6	152	ERCC5
36	MIF	75	BMP2	114	COX5A	153	FEN1
37	DCK	76	CD36	115	CRYZ	154	RAF1
38	NOS3	77	BGLAP	116	HAS3	155	CA9
39	CCNA2	78	CCL27	117	C19orf48	156	PTPN1

### 3.2. PPI network analysis and core target screening

The PPI network serves as a crucial prerequisite for the identification of core nodes that make substantial contributions [14]. Utilizing the String database, hub genes within key modules were screened through PPI network analysis, with results filtered based on a combined score of ≥0.4. Subsequently, 156 candidate targets were subjected to PPI network topology analysis in the String database to identify core targets. The outcomes of the PPI network analysis were shown in Figure 2(B), which encompassed 156 nodes and 1372 edges, were visualized using Cytoscape 3.8.0. Ultimately, 61 targets were designated as core targets due to their higher degree compared to the average level of 17.8. The top 20 targets were visualized using R4.0.3, as depicted in Figure 2(C).

### 3.3. Construction of the ingredient–target–disease network

The network depicting the relationship between formononetin, potential therapeutic targets and HCC was constructed and visualized using Cytoscape 3.8.0. This network integrated information on formononetin, potential targets and genes related to HCC to create a comprehensive network representation. The intricate interplay among formononetin, potential targets and HCC-related genes is illustrated in Figure 2(D).

### 3.4. GO and KEGG pathway analysis

**Figure 3.**
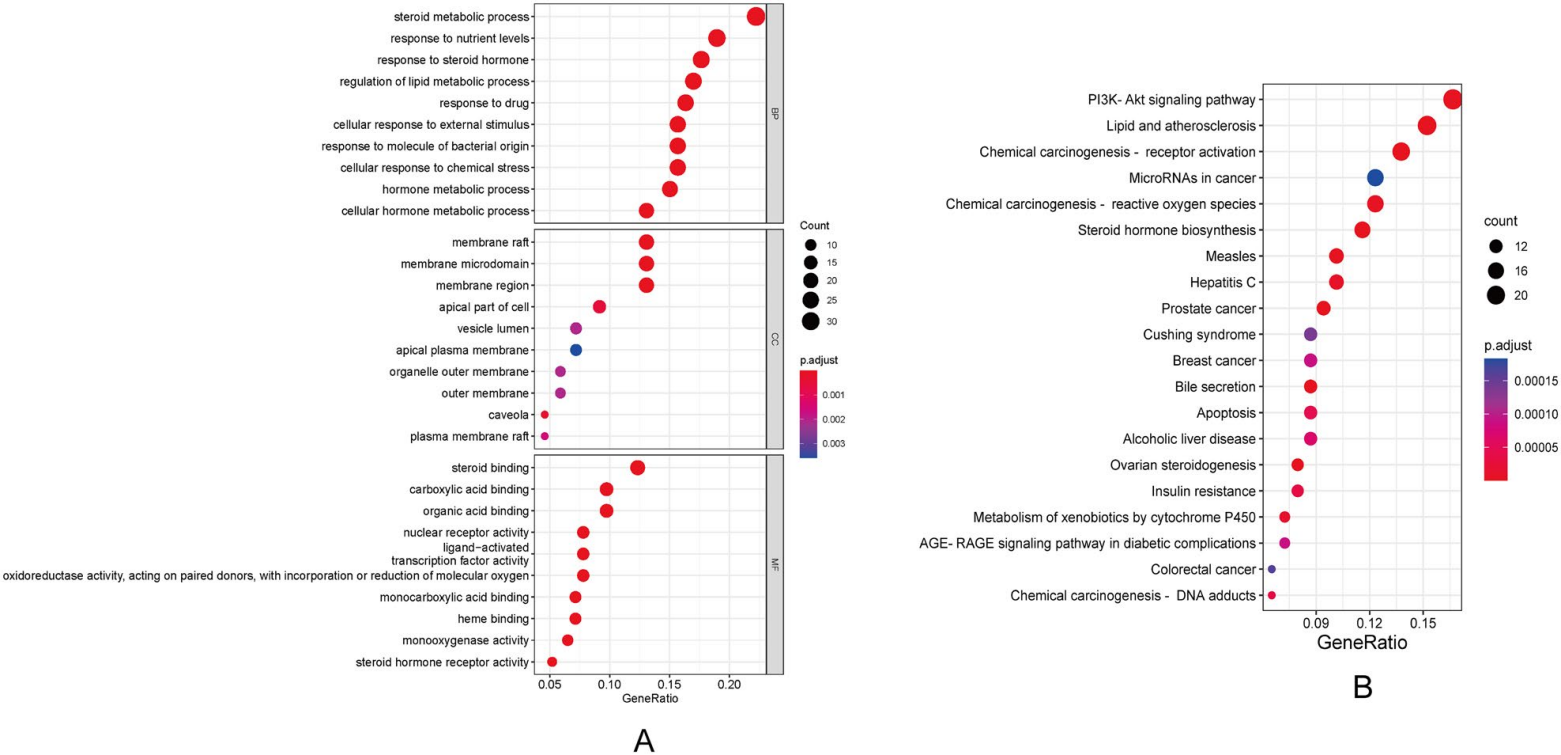
GO and KEGG terms enriched by target genes. (A) Dot plots of top 10 enriched BP, CC and MF. (B) KEGG enrichment analysis.

We used GO and KEGG pathway enrichment analysis methods to analyse the 156 overlapping targets involved in the PPI network, to further obtain the genetic function and related main signalling pathways of the target proteins. The degree of gene enrichment and the significant difference of gene enrichment are represented by gene ratio and the *p* value, respectively. The enriched terms including BP, MF and CC are shown in Figure 3(A). The items with correction *p* value <.05 were screened, and the intersection targets were enriched to 1795 BP, 43 MF and 171 CC. Among them, BP terms mainly related to steroid metabolic process, response to nutrient levels and response to steroid hormone. Enrich terms outstanding in CC were membrane raft, membrane microdomain and membrane region. MF enrich terms mainly related to steroid binding, carboxylic and binding and organic acid binding.

Furthermore, 156 target genes were classified by KEGG pathway enrichment analysis and functional enrichment using R software (R Foundation for Statistical Computing, Vienna, Austria). A total of 111 signalling pathways were enriched with a *p* value not greater than .05 were identified as significantly enriched. The top 20 enriched signalling pathways including PI3K/AKT, lipid and atherosclerosis and chemical carcinogenesis are shown in Table 2 and Figure 3(B).

**Table 2.** Top 20 pathways analysed by KEGG enrichment.

ID	Pathway	Enriched gene	*p* Value	Count
hsa0415	PI3K-Akt signalling pathway	CDK2/PDPK1/FGFR1/EGFR/PCK1/NOS3/HSP90AA1/CDK6/KDR/TP53/BRCA1/FGFR2/PIK3CG/MCL1/BCL2/FASLG/CASP9/IL4/RXRA/GSK3B/CHRM1/IL2/RAF1	1.58E − 08	23
hsa05417	Lipid and atherosclerosis	HSPA8/MAPK14/PDPK1/NOS3/HSP90AA1/MAPK8/SOD2/TP53/CD36/JUN/PPARG/VCAM1/SELE/LDLR/BCL2/FAS/CASP3/FASLG/CASP9/RXRA/GSK3B	4.36E − 11	21
hsa05207	Chemical carcinogenesis – receptor activation	ESR1/ESR2/AR/PGR/EGFR/HSP90AA1/UGT1A1/UGT1A9/UGT1A10/UGT1A7/JUN/AHR/PPARA/BCL2/TNFSF11/CYP1A2/RXRA/ADRB2/RAF1	1.74E − 09	19
hsa05208	Chemical carcinogenesis – reactive oxygen species	MAPK14/PDPK1/EGFR/AKR1C3/MAPK8/AKR1C1/SOD2/PTPN11/JUN/AHR/SOD1/CYP1A2/COX5A/CBR1/NOX4/RAF1/PTPN1	1.47E − 07	17
hsa05206	MicroRNAs in cancer	PIM1/EGFR/CDK6/PLAU/TP53/BRCA1/PTGS2/MIR375/HDAC5/MCL1/CYP24A1/BCL2/ABCC1/CASP3/SIRT1/ABCB1/RAF1	1.39E − 05	17
hsa00140	Steroid hormone biosynthesis	STS/HSD17B1/HSD11B1/AKR1C3/AKR1C1/UGT1A1/UGT1A9/UGT1A10/UGT1A7/CYP17A1/CYP11A1/CYP21A2/HSD3B2/CYP19A1/CYP1A2/HSD17B2	2.04E − 15	16
hsa05022	Pathways of neurodegeneration – multiple diseases	MAPK14/MAPK8/PTGS2/SOD1/BCL2/FAS/CASP3/FASLG/CASP9/COX5A/GSK3B/SLC6A3/NOS2/CHRM1/NOX4/RAF1	5.51E − 03	16
hsa05162	Measles	CDK2/HSPA8/CDK6/MAPK8/TP53/JUN/BCL2/FAS/CASP3/FASLG/CASP9/GSK3B/IL2/TLR9	6.41E − 08	14
hsa05160	Hepatitis C	CDK2/EGFR/CDK6/TP53/PPARA/LDLR/FAS/CASP3/FASLG/SCARB1/CASP9/RXRA/GSK3B/RAF1	2.96E − 07	14
hsa04010	MAPK signalling pathway	HSPA8/DUSP6/MAPK14/FGFR1/EGFR/MAPK8/KDR/TP53/FGFR2/JUN/FAS/CASP3/FASLG/RAF1	3.67E − 04	14
hsa05215	Prostate cancer	CDK2/AR/PDPK1/FGFR1/EGFR/HSP90AA1/PLAU/TP53/FGFR2/BCL2/CASP9/GSK3B/RAF1	6.02E − 09	13
hsa05205	Proteoglycans in cancer	ESR1/MAPK14/PDPK1/FGFR1/EGFR/PLAU/PTPN11/KDR/TP53/FAS/CASP3/FASLG/RAF1	3.43E − 05	13
hsa04976	Bile secretion	CA2/SULT2A1/NR1H4/UGT1A1/UGT1A9/UGT1A10/UGT1A7/LDLR/SCARB1/RXRA/ABCG2/ABCB1	2.22E − 08	12
hsa04210	Apoptosis	PDPK1/MAPK8/TP53/JUN/MCL1/PARP1/BCL2/FAS/CASP3/FASLG/CASP9/RAF1	2.44E − 06	12
hsa04936	Alcoholic liver disease	MAPK14/ALDH2/ADH5/MAPK8/PPARA/PPARGC1A/FAS/CASP3/FASLG/SIRT1/GSK3B/NOX4	3.84E − 06	12
hsa05224	Breast cancer	ESR1/ESR2/PGR/FGFR1/EGFR/CDK6/TP53/BRCA1/JUN/TNFSF11/GSK3B/RAF1	5.51E − 06	12
hsa04934	Cushing syndrome	CDK2/EGFR/CDK6/RASD1/AHR/CYP17A1/CYP11A1/CYP21A2/HSD3B2/LDLR/SCARB1/GSK3B	9.52E − 06	12
hsa05161	Hepatitis B	CDK2/MAPK14/CCNA2/MAPK8/TP53/JUN/BCL2/FAS/CASP3/FASLG/CASP9/RAF1	1.49E − 05	12
hsa05167	Kaposi sarcoma-associated herpesvirus infection	MAPK14/CDK6/MAPK8/TP53/PTGS2/JUN/PIK3CG/FAS/CASP3/CASP9/GSK3B/RAF1	8.86E − 05	12
hsa04913	Ovarian steroidogenesis	HSD17B1/AKR1C3/PTGS2/CYP17A1/CYP11A1/HSD3B2/LDLR/CYP19A1/SCARB1/BMP6/HSD17B2	4.90E − 10	11

### 3.5. Construction of formononetin–HCC–pathways–targets network

**Figure 4.**
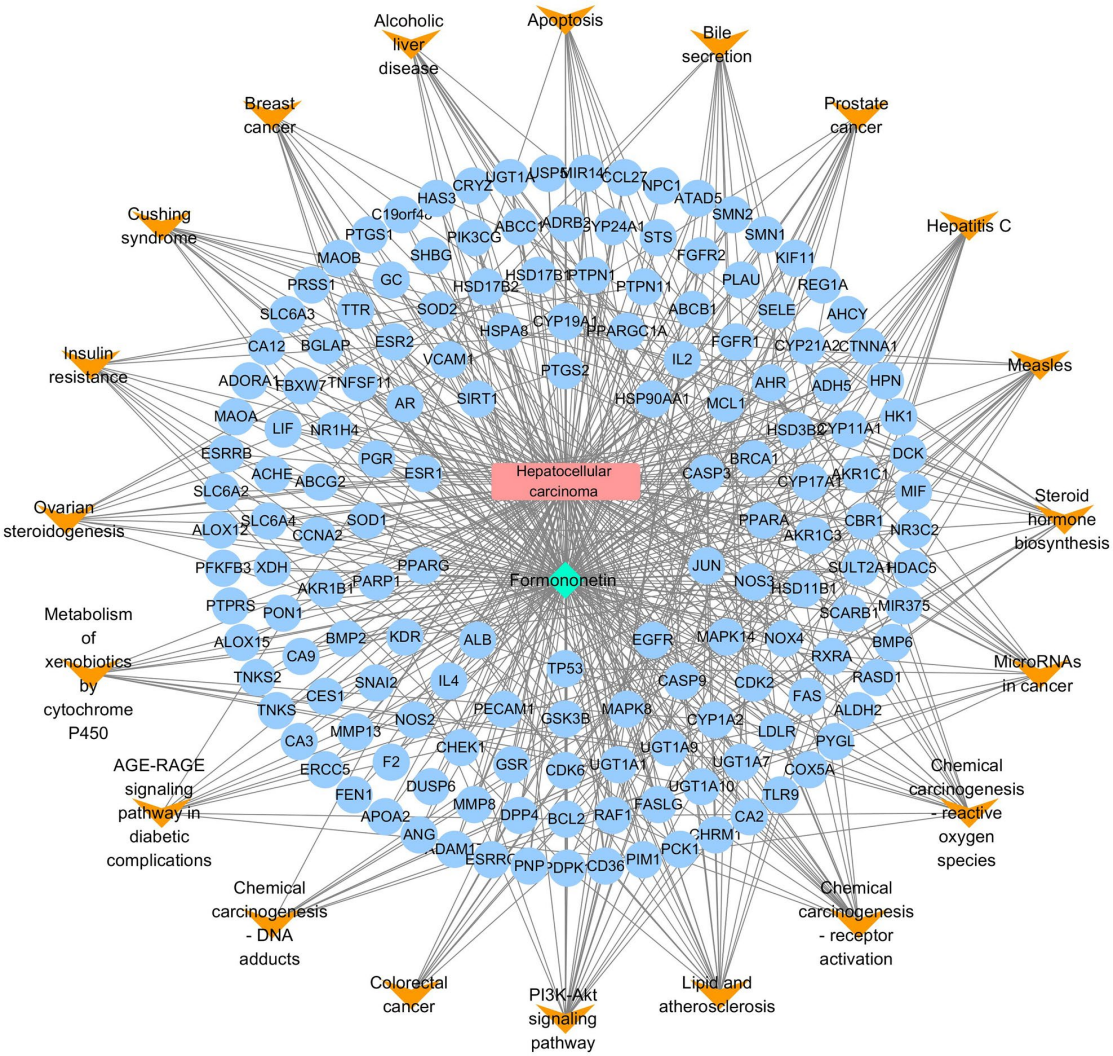
Component–disease–pathway–target network.

To visually illustrate the diverse functions of formononetin in treating HCC, the formononetin–HCC–pathways–targets network file was imported into Cytoscape 3.8.0 to generate a pathway network diagram. As shown in Table 2 and Figure 4, the most enriched signalling pathway was PI3K/AKT signalling pathway, which was related to 22 target genes including CDK2, PDPK1, FGFR1, EGFR, PCK1, NOS3, HSP90AA1, CDK6, KDR, TP53, BRCA1, FGFR2, PIK3CG, MCL1, BCL2, FASLG, CASP9, IL4, RXRA, GSK3B, CHRM1, IL2 and RAF1. The results indicated that PI3K/AKT was the key signalling pathway that formononetin improved HCC.

### 3.6. Molecular docking

**Figure 5.**
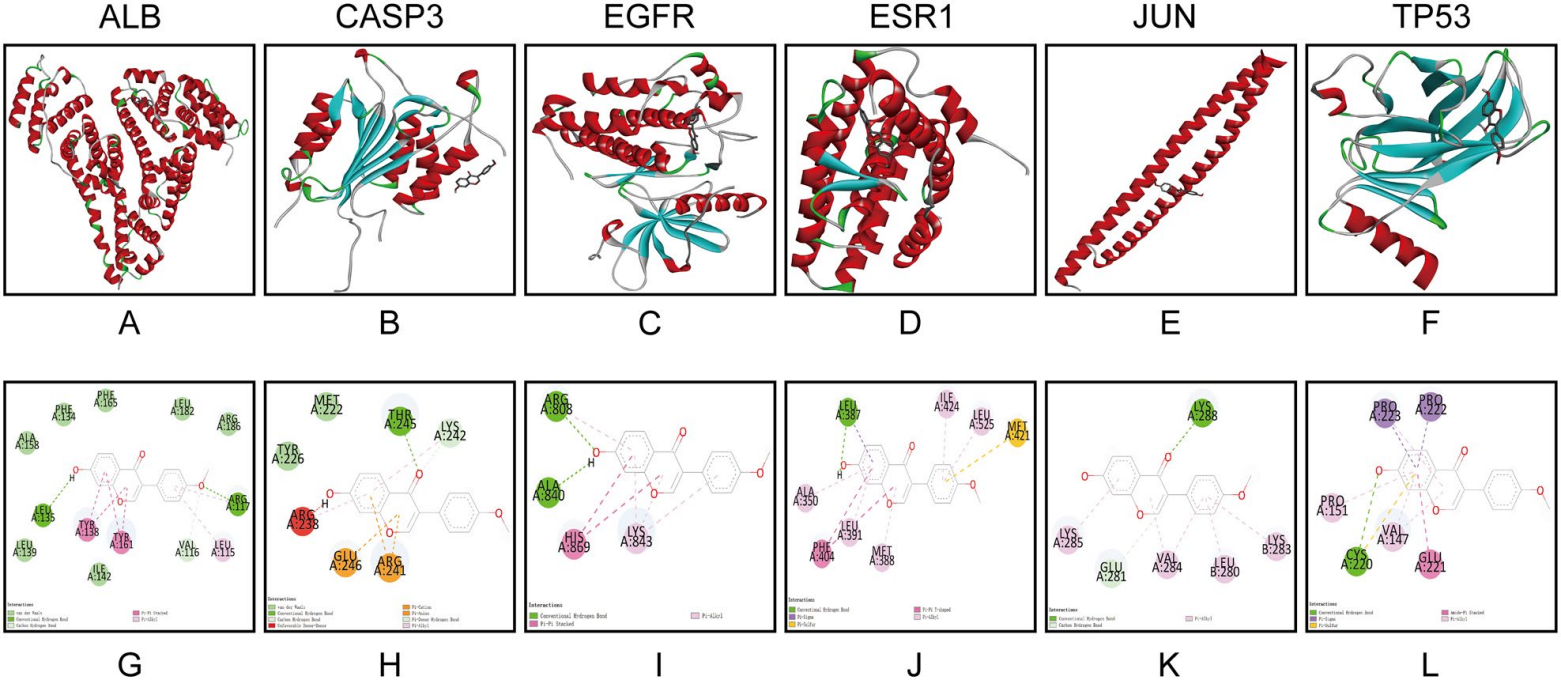
Molecular docking diagram of the formononetin and key targets. (A–F) 3D molecular docking diagram of formononetin and key targets. (G–L) 2D molecular docking diagram of formononetin and key targets.

Molecular docking is a critical approach that simulates the interaction between components and targets and can predict their binding mode and binding ability. This method effectively reduces the complexity of network research and improves the accuracy of docking [15]. In molecular docking, the more stable the binding between the chemical components and the targets, the lower the free binding energy. Six key targets including ALB, ESR1, CASP3, EGFR, JUN and TP53 were selected to molecular docking. According to the data presented in Table 3 and Figure 5(A–F), the investigation of six target proteins revealed that ALB, ESR1, CASP3, EGFR, JUN and TP53 exhibited favourable binding affinities with formononetin, with calculated values of −9.6, −9.2, −6.6, −7.0, −5.6 and −6.5 kcal/mol, respectively. The 2D docking diagram clearly showed the docking site between key targets and active compounds. The results presented in Figure 5(G–L) demonstrate that hydrogen bonds were formed between all targets and formononetin. Specifically, formononetin exhibited hydrogen bonding interactions with LEU135 and ARG117 of ALB, THR245 of CASP3, ALA840 and ARG808 of EGFR, LEU387 of ESR1, LYS288 of JUN, and CYS220 of TP53. Furthermore, formononetin was found to engage in carbon–hydrogen bonding interactions with VAL116 of ALB, LYS242 of CASP3, and GLU281 of JUN, indicating a partial mode of binding with these targets.

**Table 3.** Details of key targets and formononetin for molecular docking.

Core target	PDB ID	Binding energy (kcal/mol)
ALB	1N5U	−9.6
ESR1	6SBO	−9.2
CASP3	4JJE	−6.6
EGFR	2GS2	−7.0
JUN	5T01	−5.6
TP53	4AGP	−6.5

### 3.7. Effects of formononetin on body weight and tumour growth in mice

**Figure 6.**
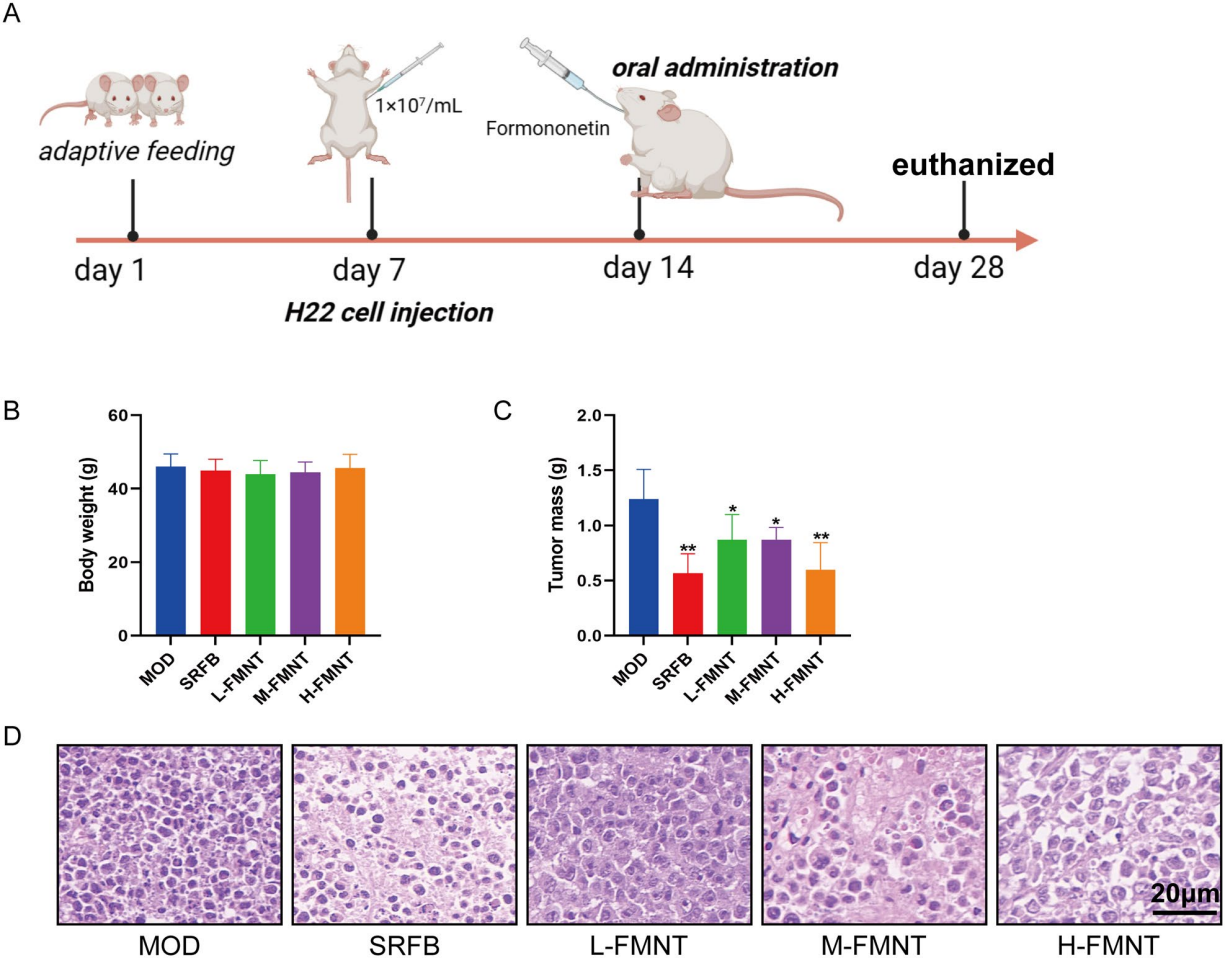
The experimental protocol (A) and effect of formononetin on (B) body weight, (C) tumour mass and (D) morpho-pathology of tumour tissues. Data are expressed as means ± SEM (*n* = 3). *p < .05 compared with the model group, **p < .01 compared with the model group.

In the animal experiment, H22 tumour-bearing mice were utilized as a model to investigate the impact of formononetin on both body weight and tumour growth. The experimental protocol, outlined in Figure 6(A), involved the injection of H22 cells into the left armpit on the 7th day, subsequent monitoring of tumour growth, and intragastric administration of formononetin and SRFB on the 14th day, with euthanasia of the mice occurring on the 28th day. The body weight of mice displayed no difference in each group in Figure 6(B). Figure 6(C) illustrates that the tumour mass of mice in the SRFB and three doses formononetin groups experienced a statistically significant decrease in comparison to the model group (*p* < .05). Among the three different doses of formononetin, the tumour mass of mice in the H-FMNT group was the lightest.

### 3.8. Histopathological examination of tumour tissues

HE staining was used to observe the histopathological change of tumour tissues in each group. In Figure 6(D), the nucleus of tumour tissues of mice in the model group is complete, dense and neatly arranged, indicating the normal proliferation of tumour cells. Compared with the model group, the cell nucleus volume in the tumour of mice in the medium-dose and high-dose groups of formononetin were significantly reduced, the number of tumour cells decreased significantly. Moreover, a part of tumour cells was destroyed, exposed cytoplasm and produced nuclear fragments. These results indicated that the proliferation of tumour cells was destroyed in the medium and high doses formononetin groups.

### 3.9. Formononetin induced tumour cell apoptosis in tumour tissues of mice

**Figure 7.**
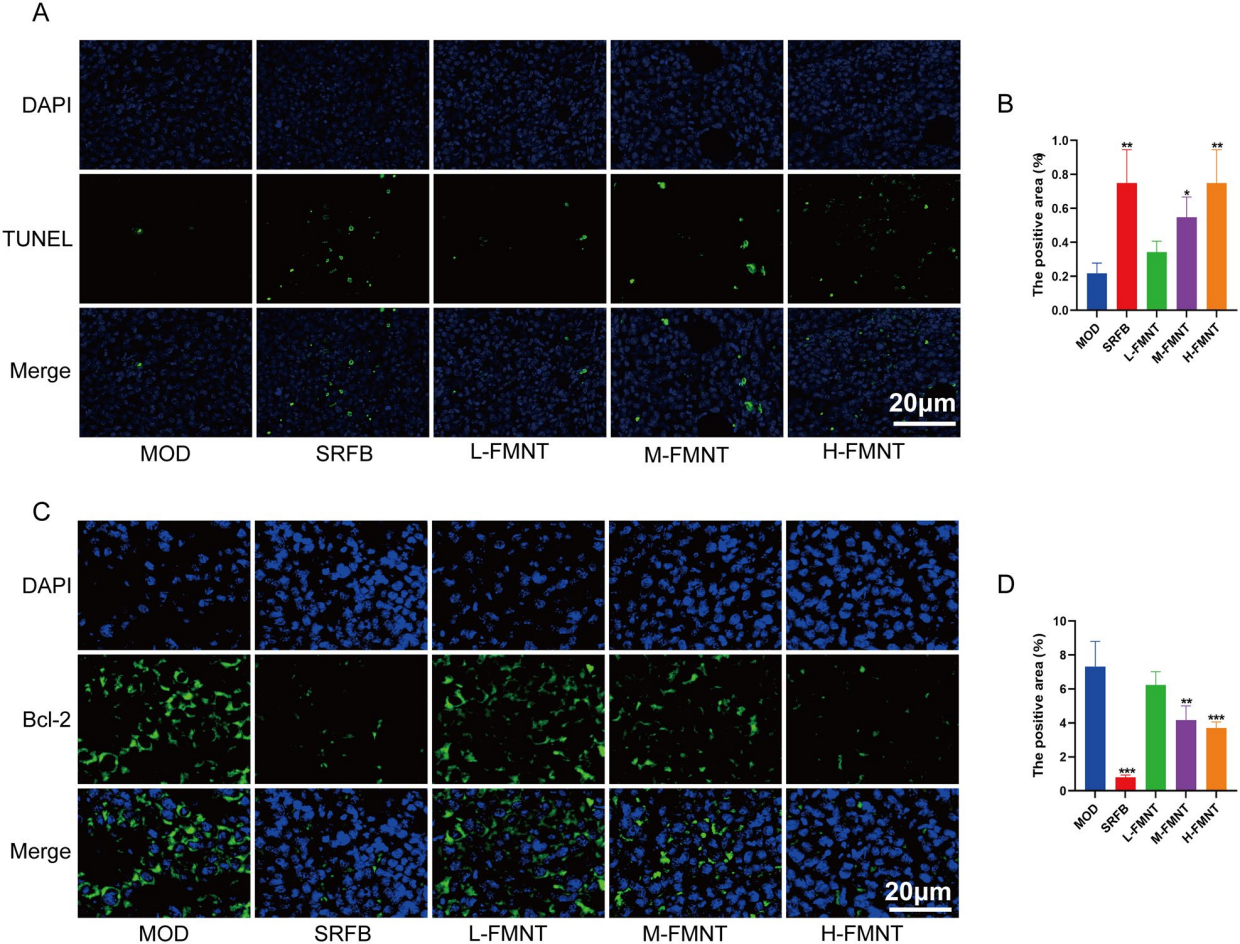
Effect of formononetin on apoptosis of tumour tissues of mice. (A, B) The apoptosis of tumour cells in tumour tissues. (C, D) The protein expression of Bcl-2 in tumour tissues of mice. Data are expressed as means ± SEM (*n* = 3). *p < .05 compared with the model group, **p < .01 compared with the model group and ***p < .001 compared with the model group.

As shown in Figure 7(A,B), TUNEL positive labelled cells are apoptotic cells (green). Compared with the model group, and the apoptosis cells of tumour tissue in the medium-dose and high-dose groups of formononetin groups were significantly increased (*p* < .05). Bcl-2 is an anti-apoptotic protein. Figure 7(C,D) shows that medium-dose and high-dose formononetin could reduce the protein expression of Bcl-2 in tumour tissues of mice (*p* < .05). Among the above results, the promotion of apoptosis of tumour cells in mice was most pronounced with high-dose of formononetin.

### 3.10. Formononetin induced cell apoptosis via PI3K/AKT signalling pathway

**Figure 8.**
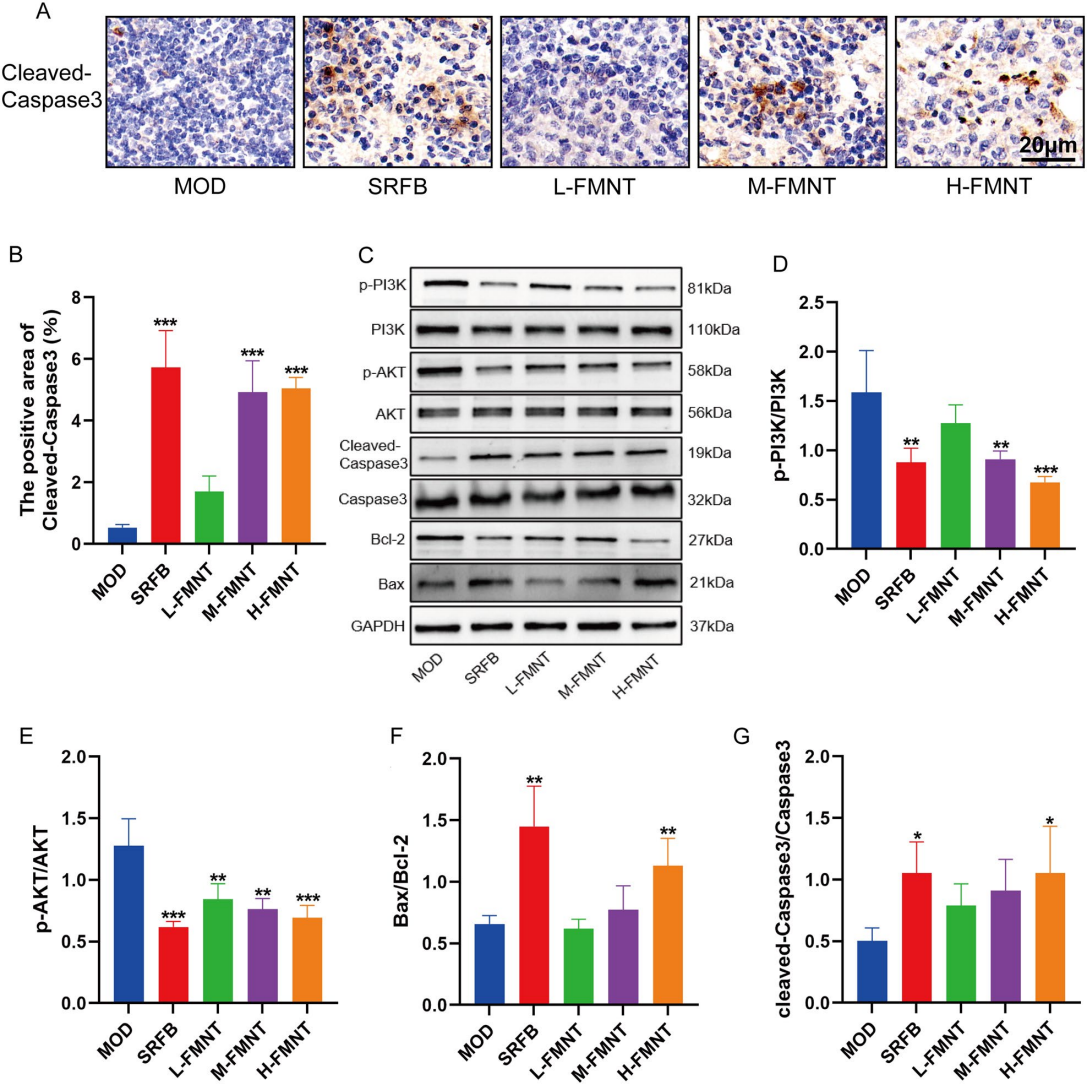
Verification of PI3K/AKT signalling pathway via Western blotting. (A, B) The protein expression of cleaved-Caspase3 in tumour tissues of mice detected by IHC. (C–G) The protein expression of PI3K/AKT signalling pathway using Western blotting. Data are expressed as means ± SEM (*n* = 3). *p < .05 compared with the model group, **p < .01 compared with the model group and ***p < .001 compared with the model group.

PI3K/AKT signalling pathway was enriched highly according to the KEGG enrichment analysis. As shown in Figure 8(A,B), medium-dose and high-dose formononetin remarkably increased the expression of cleaved-Caspase3 (*p* < .001). The results of Western blotting analysis in Figure 8(C–G) showed that formononetin markedly downregulated the protein expression of Bcl-2, p-PI3K/PI3K, p-AKT/AKT and upregulated the expression of cleaved-Caspase3 and Bax.

## 4. Discussion

Formononetin is a major isoflavone component from *Astragalus membranaceus*, a traditional Chinese medicine with function of invigorating *qi*. A previous study revealed that the inhibitory effect of formononetin on HCC cell proliferation was related to hepatocyte metabolism and cell cycle regulation-related pathways [13], the molecular mechanism of formononetin’s action of mechanism on HCC still needs to be further explored. Network pharmacology is a strategy to explore the mechanism of the interaction between the active ingredients of traditional Chinese medicine and disease from an overall perspective, and provide systematic analysis and explanation of the complex process of multi-components and multi-targets treatment of traditional Chinese medicine. This study used the network pharmacology and experimental verification to explore the potential molecular mechanism of formononetin in treating HCC from a systemic perspective.

The targets of formononetin and HCC were collected by searching and predicting in online public databases. A total of 156 targets for formononetin treating HCC were obtained by cross-mapping them with HCC-related targets in GeneCards, GEO and OMIM databases. It is worth noting that there were a total of 193 potential targets of formononetin, 156 of which were associated with HCC, indicating that formononetin has a good therapeutic effect on HCC. Furthermore, the comprehensive PPI and molecular docking analysis showed that the six key targets (ALB, ESR1, CASP3, EGFR, JUN and TP53) related to HCC had significant binding abilities with formononetin. According to previous studies, validated biotargets of ALB may be one of the main potential biomarkers for detecting HCC medically, and CASP3 is a crucial anti-HCC target [16]. Furthermore, EGFR and ESR1 have been reported to be the core therapeutic targets of HCC [17]. JUN transcription factor family is involved in regulating the proliferation, migration and apoptosis of HCC, which is closely related to the occurrence and development of HCC [18]. Moreover, TP53 mutation is one of the most common genetic changes in HCC [19]. Among that, CASP3, JUN and TP53 are apoptosis regulatory factors, indicating that formononetin’s treatment of HCC closely related to apoptosis.

GO functional enrichment analysis was used to explore the pharmacological mechanism of formononetin in treating HCC. The results revealed that the GO enrichment pathways of HCC mainly involved in the regulation of steroid metabolic process, membrane raft and steroid binding. KEGG analysis showed that the targets were involved in multiple pathways, including PI3K/AKT signalling pathway, lipid and atherosclerosis, chemical carcinogenesis – receptor activation, chemical carcinogenesis – receptor activation and apoptosis, and the PI3K/AKT signalling pathway is the most enriched signalling pathway. Numerous studies have confirmed the abnormal activation of the PI3K/AKT signalling pathway in HCC [20]. The activation of AKT can regulate the proliferation, differentiation, invasion, migration and apoptosis of cancer cells by regulating downstream protein factors, such as B-cell leukaemia lymphoma-related protein gene (Bax), anti-apoptosis protein Bcl-2 and cysteine protease [21,22]. These results indicated that formononetin has anti-HCC effects, the mechanism of action of formononetin on HCC worthy of further exploration.

It was determined that formononetin has the potential ability to modulate HCC through various targets and pathways by using network pharmacological analysis, so we conducted further *in vivo* validation. The animal experiments showed that formononetin could effectively inhibit the growth of tumour in mice after being injected H22 cells. Mice in formononetin groups displayed lower tumour mass than that of model group. The results of histopathological examination of tumour tissues revealed that formononetin reduced the cell nucleus volume in the tumour and the number of tumour cells. Furthermore, the *in silico* results revealed that formononetin treats HCC through regulating PI3K/AKT signalling pathway mediated apoptosis. Our findings indicated that formononetin caused a notable downregulation in Bcl-2, p-PI3K, p-AKT and upregulation in Caspase3 and Bax, indicating that the inhibition of the PI3K/AKT pathway is correlated with increased apoptosis in HCC. Therefore, we speculated that formononetin may possess a protective effect on HCC by enhancing apoptosis via the PI3K/AKT pathway. However, there are still some limitations in this study, the use of activator and inhibitor on the PI3K/AKT pathway was lacked in *in vivo* experiment, thus additional studies are required to further delineate the mechanistic actions of formononetin on HCC.

## 5. Conclusions

This study provides evidences supporting the efficacy of formononetin in treating HCC, showing that the therapeutic effect of formononetin on HCC is dependent on dosage and most effective at higher doses. Furthermore, the research suggests that mechanism of action of formononetin in treating HCC involves apoptosis mediated by the PI3K/AKT signalling pathway. The study provided a basis for formononetin in the treatment of HCC, and provided a new direction for the treatment of HCC.

## Data Availability

The original data of this study will be made available by contacting the corresponding author (bzsyhsp2023@163.com).
